# Validation of Hepatocellular Carcinoma Experimental Models for TGF-β Promoting Tumor Progression

**DOI:** 10.3390/cancers11101510

**Published:** 2019-10-09

**Authors:** Serena Mancarella, Silke Krol, Alberto Crovace, Stefano Leporatti, Francesco Dituri, Martina Frusciante, Gianluigi Giannelli

**Affiliations:** 1 National Institute of Gastroenterology, “S. de Bellis” Research Hospital, Castellana Grotte, Bari 70013, Italy; serena.mancarella@irccsdebellis.it (S.M.); alberto.crovace@irccsdebellis.it (A.C.); francesco.dituri@irccsdebellis.it (F.D.); martina.frusciante@aferetica.com (M.F.); 2 CNR NANOTEC—Istituto di Nanotecnologia, Lecce 73100, Italy; stefano.leporatti@nanotec.cnr.it

**Keywords:** hepatocellular carcinoma, TGF-β, tumor microenvironment, Galunisertib

## Abstract

Transforming growth factor beta (TGF-β) is a pleiotropic cytokine with dual role in hepatocellular carcinoma (HCC). It acts as tumor-suppressor and tumor-promoter in the early and late stage respectively. TGF-β influences the tumor-stroma cross-talk affecting the tumoral microenvironment. Therefore, inhibiting the TGF- β mediated pathway alone and/or in combination with chemotherapeutics represents an important therapeutic option. Experimental models to dissect the role of TGF-β in HCC tumor progression as well as the effectiveness of specific inhibitors are tricky. HCC cell lines respond to TGF-β according to their epithelial phenotype. However, the mesenchymal and more aggressive HCC cell lines in vitro, do not develop tumors when transplanted in vivo, thus hampering the understanding of molecular pathways that dictate outcome. In addition, in this model the native immune system is abolished, therefore the contribution of inflammation in hepatocarcinogenesis is unreliable. Different strategies have been set up to engineer HCC animal models, including genetically modified mice, chemically induced HCC, or hydrodynamic techniques. Patient-derived xenograft is currently probably the most fascinating model, keeping in mind that models cannot mirror all the reality. In this context, we discuss the different available HCC mouse models including our experimental model treated with inhibitor of TGF-β receptor Type I kinase (Galunisertib) and a potential role of exosomes in TGF-β moderated tumor progression of HCC. Unfortunately, no positive results were obtained in our treated orthotopic model because it does not reproduce the critical tumor-stroma interactions of the HCC.

## 1. Introduction

**Figure 1:**
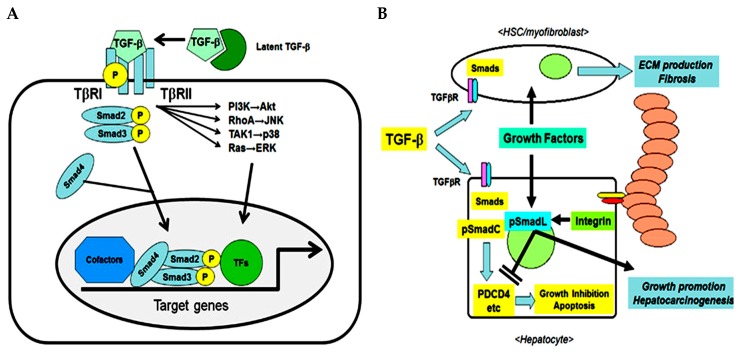
Transforming growth factor (TGF)-β pathway in fibrotic liver which can lead to hepatocellular carcinoma. (**A**) TGF-β as trigger of cellular response (**B**) TGF-β in the cross-talk between hepatocytes and microenvironment (Under Creative Commons Attribution (CC BY) license from: Ozaki et al. [3].

The transforming growth factor (TGF) family consists of three multifunctional cytokine forms (TGF-β1, TGF-β2, TGF-β3) synthetized as latent precursors and secreted as homodimers or heterodimers. Briefly, TGF-β ligand binds the Type II serine/threonine kinase dimeric receptor (TGFβR2) recruiting and inducing phosphorylation of the Type I dimeric receptor (TGFβR1). In this way it forms a tetrameric complex which phosphorylates and activates the down-stream SMAD proteins such as SMAD2/3 and SMAD4 that in turn can have an inhibitory or stimulatory effect [1] (Figure 1A). TGF-β1 is highly expressed in malignant tumors, including hepatocellular carcinoma (HCC). In combination with other growth factors/cytokines such as hepatocyte growth factor (HGF), platelet-derived growth factor (PDGF), vascular endothelial growth factor (VEGF), tumor necrosis factor-α (TNF-α), interleukins (IL) and interferons (IFN) released from various types of liver cells, it participates in apoptosis, inflammation, cell proliferation, chronic inflammation, angiogenesis and host resistance mechanisms [2], fibrosis/remodeling of tissues and the development/progression of HCC [3].

In the initial phase of hepatic injury, TGF-β1 inhibits hepatocyte proliferation and stimulates fibroblast proliferation by inducing extracellular matrix (ECM) gene expression to repair damaged tissue. However, when tissue repair becomes a continuous process as in the case of persistent hepatic virus infection, the production of ECM increases (fibrosis), leading to the formation of cirrhosis that predisposes the liver to HCC (Figure 1B) [3]. Whereby, TGF-β activity depends strongly on the interaction with different cell types or cytokines and can have autocrine or paracrine effects [4].

In the following review we will highlight the role of TGF-β pathway in HCC in its soluble form as well as exosomal cargo and how well this role is reflected by the respective mouse models.

### 1.1. Regulation Strategies of TGF-β Signaling in HCC Cells

TGF-β is well documented as a pleiotropic cytokine with a dual role in hepatocarcinogenesis. Indeed, there is a strong evidence that it works as an inducer of apoptosis and acts as a tumor-suppressor regulator in the early stages of the carcinogenesis, but also then becomes a pro-tumorigenic factor once the tumor cells are able to overcome its suppressing effects, thereby stimulating tumor proliferation and survival. Nevertheless, the switch from tumor-suppressor to pro-oncogenic is not yet well understood.

Recently, Coulouarn et al. [5] have shown that a distinct signature of TGF-β gene expression observed in cell lines and in patients with HCC results in significant differences in tumor invasiveness, time before relapse, and hence long-term survival of the patient. HCC patients with pro-oncogenic TGF-β signature tumors have a considerably shorter mean survival time and increased risk of tumor recurrence compared to patient tumors with the TGF-β early stage signature. The early stage signature is observed also in the following cell lines: PLC/PRF/5, Hep3B-TR, Hep3B, Hep40, HUH7, HepG2, HUH6, and SNU 449. HCC tumors with late stage TGF-β signature, which is found also in HLE, SK-Hep1, HLF cell lines, are characterized by an overexpression of positive cell cycle regulators, genes involved in angiogenesis consistent with a more aggressive phenotype related to a poor clinical outcome. In the transition between early and late stage, TGF-β acts as a powerful stimulator of epithelial-mesenchymal transition (EMT). EMT is a biological process that induces a de-differentiation of the polarized, epithelial to the fibroblastoid, mesenchymal phenotype. This is caused by a loss of cell–cell adhesion and therefore of the apico-basal polarity with consequent remodeling of the cytoskeleton. Furthermore, the tumor cells lose the expression of epithelial markers such as E-cadherin and acquire the expression of mesenchymal proteins such as N-cadherin, α smooth muscle actin (α-SMA) and EMT transcription factors, Snail (SNA1), Slug (SNA2), Twist and ZEB. This re-programming induces an increase in motility and invasiveness promoting intra- and extrahepatic metastases [6].

**Figure 2:**
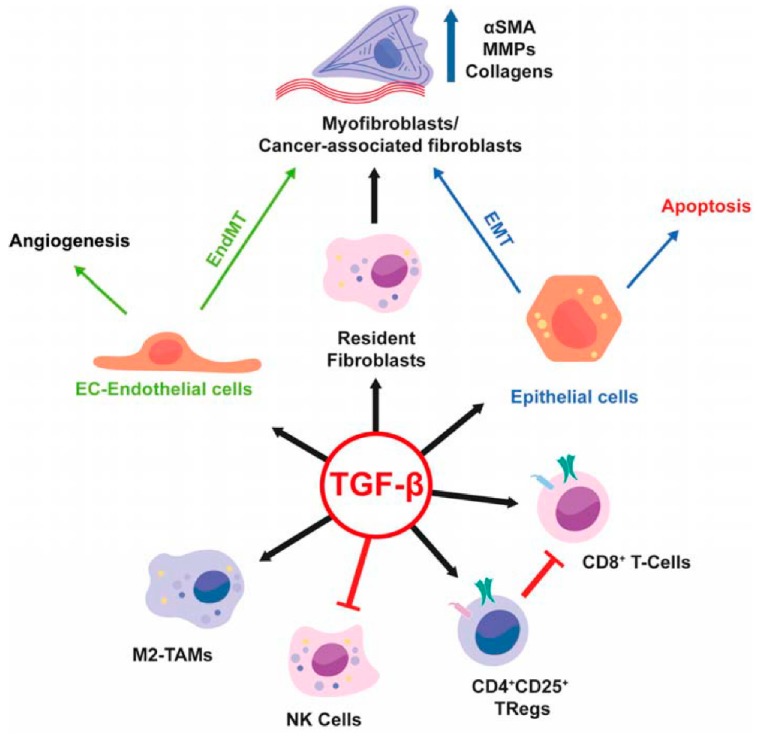
Role of TGF-β in the tumor microenvironment (Under Creative Commons Attribution (CC BY) license from: Caja et al. [7]).

An area of intense investigation is the cross-talk between tumor cells and surrounding microenvironment as well as the role of the microenvironment in the regulation of tumor progression of HCC (Figure 2). The presence of TGF-β in the tumor microenvironment induces in chronic liver lesions the activation of quiescent hepatic stellate cells (HSC) in myofibroblasts. These produce an increase in the production of angiogenic factors, recruitment of macrophages and fibrogenic α-smooth muscle actin (α-SMA) which drives the progression of fibrosis with subsequent cirrhosis [7]. During liver tumorigenesis, neoplastic cells recruit fibroblasts through various types of growth factors and cytokines and become cancer-associated fibroblasts (CAFs) which provide an important source of ECM proteins and other fibrogenic factors.

CAFs through TGF-β stimulation exert a promoting effect in the tumor, conditioning the behavior of neighbor cells with the secretion of other growth factors, cytokines, and ECM. Therefore, CAFs play a decisive role in tumor progression because they make the microenvironment immunosuppressive and drug resistant [8].

Directly, CAFs secrete TGF-β that triggers EMT in HCC cells inducing the expression of stemness-related genes including CD44 up-regulation. Indeed, it has been observed that the mesenchymal phenotype is closely related to the high expression of the CD44 marker, with enhancement of aggressiveness and migratory/invasive capacity such as in cancer stem cells. Therefore, CAFs act as a paracrine niche to support stemness [9] and they are responsible for tumor re-initiation and chemoresistance in patients with HCC [10]. CAFs produce several growth factors including a significant amount of HGF, epidermal growth factor (EGF), fibroblast growth factor, Wnt families and cytokines, such as stromal-derived factor (SDF)-1α, IL-6 [11,12]. In addition, in co-culture with HCC cells, CAFs induced by TIMP-1 inhibit tumor apoptosis by increasing Bcl-2/BAX ratio through SDF-1/CXCR4/PI3K/AKT signaling activation in HCC [13]. Besides, it has been shown that CAFs contribute to hepatic regulatory T (Treg) cell induction and recruit myeloid derived suppressor cells, which are able to inhibit the activity of cytotoxic lymphocyte cells preventing their infiltration into the tumor [14,15,16]. In this way, CAFs promote HCC by reducing immune surveillance creating an immunosuppressive microenvironment.

Interestingly, Porcelli et al. showed the key role of mast cells in the combination resistance of gemcitabine/nabpaclitaxel (GEM/NAB) in pancreatic ductal adenocarcinoma reducing apoptosis, activating TGF-β signaling and promoting tumor invasion. This inhibition was then restored by Galunisertib (LY2157299) a small molecule that specifically inhibits TGF-β receptor Type I kinase and the phosphorylation of SMAD2 reducing the activation of the canonical pathway. These findings suggest that cancer-released TGF-β acts as a powerful player to escape cancer therapies [17].

In fact, numerous strategies have been developed to inhibit TGF-β signaling including anti-oligo, monoclonal antibodies, or small molecules [18].

Recently, microRNAs (miRNAs) have been shown to act as regulators of fibrosis and HCC by interfering with the TGF-β pathway. In this regard, it was observed that miR-9-5p could inhibit TGF-β1-induced HSC activation and its loss is associated with its methylation status in liver fibrosis [19]. MicroRNA-34a-5p (miR-34a-5p) is another miRNA whose expression is significantly reduced in patients with liver fibrosis due to hepatitis B virus infection as well as in HCC. The same effect is observed in mice with HCC induced by CC14 (tetrachloromethane) injection. In particular, the TGF-β1 SMAD2/3 pathway is significantly increased in CC14-induced mice as compared to normal mice, while the TGF-β1 inhibitor (SB431542) significantly attenuated liver fibrosis and TGF-β1 SMAD2/3 [20]. Therefore, it appears that miR-34 overexpression improves the development and progression of hepatic fibrosis by targeting SMAD4 and regulating the TGF-β1 SMAD2/3 pathway. On the contrary, the miR-663a is a tumor-suppressor and plays a substantial role in inhibiting HCC proliferation, invasion and tumorigenesis by regulating TGF-β1 in vitro and in vivo [21]. These observations indicate that the regulation of these miRNAs can benefit the treatment of fibrosis and HCC.

However, because of the dual dichotomic role of TGF-β both protumorigenic and oncoprotector, and the different activity displayed by TGF-β in the distinct tissues, limits the effectiveness of these inhibitors in clinical studies is limited. This is the case of the monoclonal antibody D10 which showed efficacy in inhibiting the tumoral growth of breast cancer in mice but was failed in treatment of HCC likely because of restoring of the TGF-β receptors on the cancer cellular surface [22]. In the same study, the authors also showed that exogenous TGF-β1 in vitro reorganizes the structure of HepG2 cells making them flat and larger, therefore comparable to cancerous nodules. Furthermore, TGF-β1 reduces the expression of E-cadherin in cell–cell contact, hence increasing invasiveness. These effects are reversed by the treatment with Galunisertib. Another work reported that the expression of CD133 and Vimentin was upregulated by treatment with the TGF-β receptor antagonist SB431542 [23]. Additionally, it was observed by Rani and colleagues that treatment with Galunisertib in vitro modulates the expression of stemness-related genes in invasive HCC cells (HLE and HLF) reducing the expression of CD44 and THY1 and consequently results in decrease in colony formation, liver spheroids and invasive growth capacity in vitro and in ex vivo human HCC samples [24,25]. Additionally, Next-Generation Sequencing-based massive analysis of cDNA ends revealed increased SKIL and PMEPA1 mRNA expression in HCC tumor tissues compared to controls that positively correlate with TGF-β1 mRNA concentrations in HCC tissues. These genes were strongly down-regulated by treatment with Galunisertib [26]. Finally, these experimental observations confirmed the efficacy of Galunisertib, a promising drug recently under clinical investigation for treatment in HCC patients, in vitro for HCC cells and ex vivo on patient samples, a promising drug recently under clinical investigation for treatment in HCC patients.

### 1.2. Mouse as a Translational Model for TGF-β Action in HCC

Animal models have an important role in studying tumor development and progression and to understand the molecular biological mechanism of diseases or to validate new anticancer drugs.

To study HCC, the mouse (*Mus musculus*) is one of the most suitable animal models for its small size and for ease of breeding and growth of large populations of genetically identical animals in a short time. Different approaches such as chemically induced liver tumors, implantation of syngeneic material or xenografts or genetically engineered mouse models [27,28] are used to study HCC so that the model reproduces the human hepatic lesions at histological and molecular levels and allow evaluation of the action of new therapeutic drugs.

There are several well-established cancerogenic compounds to chemically induce liver injury and as a consequence HCC such as genotoxic compounds. One example is N-nitrosodiethylamine (DEN) that induces DNA structural changes. Alternatively HCC can be induced by non-genotoxic carcinogens such as phenobarbital, thioacetamide, di(2-ethylhexyl)phthalate, methapyrilene, carbon tetrachloride (CCl4), and tamoxifen that act by destructing or damaging the cells increasing the risk of genetic errors and stimulating the evolution of cells in malignant neoplasms by influencing proliferation, differentiation and apoptosis mechanisms [28,29,30].

For implantation, one can distinguish allogenic or allograft and xenogenic or xenograft models depending on the host species or the origin of the tumor. Allografts are established by injecting of HCC cell lines or tumor fragments or 3D structures (e.g., organoids) derived from the same species in immunocompetent mice (not necessarily syngeneic). This model is extremely useful to study the effects of immunotherapies and to observe the inflammatory response. The xenograft model is established by inoculating cell lines or tumor tissues/organoids derived from different species in immunodeficient mice models [31]. This is an approach that allows the formation of metastasis and gives a better overview of the development of the tumor during therapies [32]. However, in this case the interaction between neoplastic cells and the surrounding microenvironment could be lost. This shortcoming is addressed by the implantation of patient-derived xenografts (PDXs) that allow the investigation of cellular responses in an environment comparable to the patient tissue. Another limitation of HCC cell lines implantation is that they are not always tumorigenic in vivo. Mazzocca et al. observed that HuH7 and HLE cell lines were tumorigenic in vivo only with Lysophosphatidic acid receptor overexpression [29]. Even though HCC models are often used for drug screening the results observed in mice models cannot always be repeated in cancer patients.

The graft implantation can be performed in different sites of inoculum. Moreover, the allograft and xenograft models can be sub-cataloged as heterotopic or orthotopic models. In case of heterotopic implantation, cells, tumor fragments or organoids are implanted in a site different from that of explantation [32]. For example, subcutaneous grafts in nude immune deficient mice are mostly used as heterotopic model due to ease of access for monitoring the tumor growth. However, the difference in microenvironment can cause an anomaly in the behavior of tumor cells giving false positive results [32]. The importance of implantation site was underlined in studies by Basu and Herlyn [33] and Speroni et al. [34]. They noted that the dorsal foot as implantation site for HCC cells is preferable as it mimics better the conditions for aggressive tumor progression in terms of cellular invasion and local immune reaction. Moreover, only those implanted cell masses developed into advanced tumors with lung metastasis [34]. This is usually not observed for subcutaneous grafts in the hind leg of mice. Orthotopic models more closely mimic the conditions of a tumor since they are growing under the organ-specific conditions such as pH, cell density, ECM conditions. However, it has to be mentioned that the orthotopic models also show differences in the microenvironment in particular for B cell infiltration, as was shown by Spear et al. [35] for pancreatic cancer. To address the shortcomings and advantages of both models, Rao et al. [36] proposed an interesting comparison between common animal models including intrahepatic, intrasplenic, subcutaneous inoculation to implant murine or human tumor cells or tumor fragments to develop HCC in C57BL6 mice. They showed a superior tumor formation from the direct implantation of tumor fragments in the liver. The drawback of this approach is the monitoring of tumor growth in the treated living animal as it does not show any symptoms. To calculate the increase in tumor mass requires repeated imaging with contrast agent or GFP-modification of the implanted cells and anesthesia.

Finally, HCC is studied in genetically modified mouse models, including knockout or transgenic mice, useful for demonstrating the oncogenic potential or tumor-suppressor potential of target genes, and that are designed to mimic the pathophysiological and molecular characteristics of tumor. Recently, the hydrodynamic tail-vein injection was established as a new method for general investigation of hepatic gene regulatory elements or drug response. For this, a large volume of DNA solution is administered into the tail vein. The bulk injection of a large volume into the blood circulation causes a temporary blood pressure increase and a cardiological dysfunction that can be linked to a right ventricular dilation. Sawyer et al. [37] observed that even a small volume can cause this condition. The surplus liquid causes an enlargement the liver an enlargement that can be related to the speed of injection and the volume of the solution [38]. This process can be used to study HCC as demonstrated by Chen and Calvisi. They combined hydrodynamic tail administration with delivery of knockout genes to test different oncogenes (in single dose or combinations) and evaluate the effects on development of liver tumors. A drawback of intrahepatic tumors is the monitoring of the process, e.g., the growth and distribution of tumor cells or development of fibrosis or cirrhosis common conditions in human hepatocarcinogenesis without sacrificing the animals at different timepoints [39].

In order to complete the list of HCC models for the study of involvement of the TGF-β pathway we summarize the previously discussed models and some additional mouse models for HCC (Table 1).

### 1.3. Inhibition of TGF-β in Experimental Mouse Models

Given the relevant role of TGF-β in promoting the progression of HCC, in vivo models are considered an important tool for the development of a TGF-β inhibitory strategy for HCC patients. For instance, the well characterized immunodeficient xenograft mouse model was not suitable to investigate the effectiveness of TGF-β pathway inhibitors after positive engraftment of the tumors. The reasons that underlie this problem are not yet clear. In the following, we report the results for an orthotopic HCC mouse model induced by intrahepatic injection of HepG2-Luc cell in CD-1 and NOD/Shi-Scid/IL-2Rγ^null^ (NOG) strains followed by treatment with Galunisertib. The study has been conducted at the Biogem Animal House (Avellino, Ariano Irpino, Italy) in accordance with the ethical standards and according to national and international guidelines and has been approved by the authors’ institutional review board (Organism for Animal Wellbeing—OPBA). All animal experiments were carried out in accordance with Directive 86/609 EEC enforced by Italian D.L. n. 26/2014 and approved by the Committee on the Ethics of Animal Experiments of Ministero della Salute—Direzione Generale Sanità Animale. Animals were sacrificed if in severe clinical conditions in order to avoid suffering.

Firstly, we quantified the concentration of LY2157299 in plasma of female CD-1 mice by a liquid chromatographic method, in order to define the concentration for the therapeutic window. Therefore, the drug was administered orally and given in two formulations. Group 1 (15 mice) received Galunisertib as suspension (75 mg/kg) prepared by mixing Galunisertib with 1% Carboxymethylcellulose, 0.5% Sodium Lauryl Sulfate, 0.085% Povidone, followed by sonication in a water bath sonicator or with a probe sonicator until a fine suspension with a final concentration of 7.5 mg/mL was achieved. Group 2 (15 mice) received Galunisertib as solution (150 mg/kg solution) prepared by dissolving the drug in 10% of dimethylsulfoxide (DMSO), and then adding sequentially polyethylene glycol (PEG)400 (30%), ethanol (EtOH) (10%), saline (30%) and HCl 0.01 M (20%) at a final pH of ~6 under mixing to a final concentration of 15 mg/mL. We observed that the new formulation, the 150 mg/kg Galunisertib solution, presented as a completely clear solution while in the old formulation (75 mg/kg) Galunisertib was only moderately soluble.

The volume of administration was 10mL/kg for single treatment. Physical appearance, behavior as well as general and local clinical signs of the mice were monitored throughout the experiment. No acute toxicity was observed after oral administration of Galunisertib. At predefined time points (0.5, 1, 2, 4, 8 h after administration), the animals were deeply anesthetized by isoflurane followed by a complete retro-orbital bleeding. The blood was collected in heparinized tubes and centrifuged at 6000× *g* for 5–10 minutes within 15 minutes after collection. The plasma was transferred to tubes and stored at −20 °C until analysis.

To quantify the concentration of Galunisertib, plasma was measured by a HPLC/Fluorimetric (HPLC-FL) method. Briefly, the linear calibration curve in the examined concentration range of 0.05 to 2.0 µg/mL showed a *R^2^* ≥ 0.99 with the limit of quantitation represented by the lowest point on the calibration curve (Table 2).

Accuracy and precision was determined from the repeated analysis of quality control samples. The inter-assay precision and accuracy were lower than 5% and within −6.42 and 1.68%, respectively (Table 3) indicating a good performance of the method in terms of linearity, accuracy and precision. In addition, the sensitivity of this assay enabled detailed characterization of Galunisertib concentrations to 8 hours post oral administration for the two different formulations.

**Figure 3:**
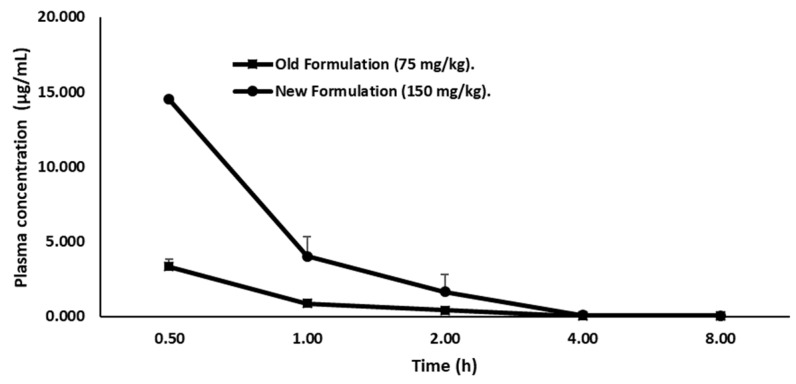
Plasma concentration versus time profiles of Galunisertib following New Figure 150 mg/kg; squares) or Old Formulation (75 mg/kg; circle) oral administration to mice. Values are mean ± SD of three animals/time points.

Plasma concentration-time profile of Galunisertib is shown in (Figure 3).

The pharmacokinetic parameters (Kelim, T1/2, and Tmax.) represented as mean ± SD of three animals/time for both Galunisertib formulations were determined using the MKModel by Nick Holford software Version 4, (Biosoft, Ferguson, MO, USA) or equivalent pharmacokinetic programs (Table 4).

The formulation with 150 mg/kg Galunisertib achieved a C_max_ and AUC_last_ of 19.01 µg/mL and 15.48 µg/h/mL, respectively. Therefore, this formulation showed a C_max_ and AUC_last_ 2.5-fold higher than the 75 mg/kg Galunisertib suspension (C_max_ = 3.11 µg/mL and AUC_last_ = 3.33 µg/h/mL) considering the dose correcting factor. Consequently, the 150 mg/kg Galunisertib solution was administered either via the parental or the enteral route.

### 1.4. Efficacy Study of Galunisertib in Orthotopic Mouse Model

To evaluate in vivo the effect of Galunisertib (LY2157299) on tumor growth rate we used an orthotopic model: HCC was induced by intrahepatic injections of 1 × 10^6^ HepG2-Luc cells in 7–8 weeks old, female NOG mice. The inoculated animals presented a clear engraftment of the liver cancer. The tumor growth was monitored weekly with IVIS Spectrum (PerkinElmer) through intraperitoneal administration of D-Luciferin (Sigma, 150 mg/10 mL/kg) until the end of the experiment.

Two weeks after the injection of HepG2-Luc cells, 45 mice were selected based on the bioluminescence signal and randomized into three groups of 15 animals. The first group (control group) was treated with 10 mL/kg of a of 15 mg/mL vehicle solution (10% DMSO, 30% PEG400, 10% EtOH, 30% saline solution, 20% HCl 0.01 M, final pH 6.0). The second group (GP2) received 10 mL/kg Galunisertib solution via gastric probe (OS) and in the third group of mice (GP3) the drug was administered intravenously (IV) with 5 mL/kg Galunisertib encapsulated in polymeric nanocapsules. This nanoencapsulation was used to optimize the formulation in order to maximize therapeutic efficiency and minimize systemic toxicity. The nanocapsules were prepared from biocompatible and biodegradable polymers as described previously [46].

**Figure 4:**
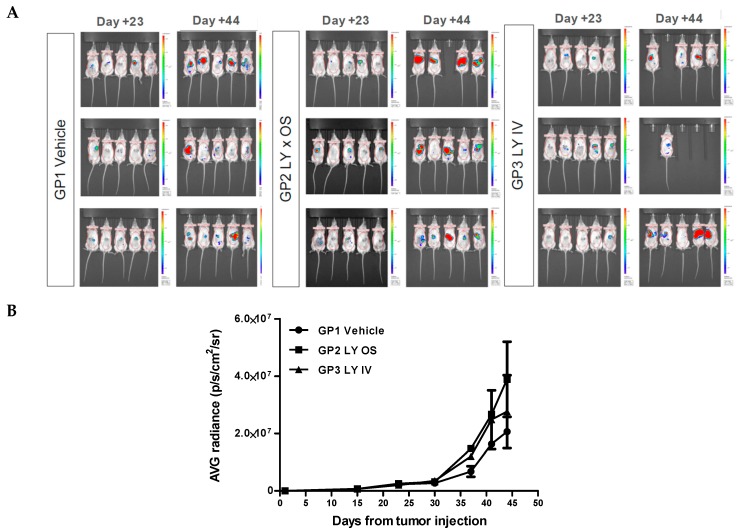
Treatment of NOD/Shi-Scid/IL-2Rγ^null^ Common gamma Ray (NOG) mice with luciferin-labeled hepatocellular carcinoma (HCC) with vehicle or two Galunisertib preparations. (**A**). Bioluminescence images of NOG mice prior injected intrahepatically with HepG2-Luc cells at Day 23 and 44 after vehicle injection (GP1), or Galunisertib administration via gavage (GP2) or intravenous route (GP3). (**B**) Average Radiance (IVIS) versus time after tumor inoculation. Data were averaged over the animals which reached the end of experiment (GP1: *n* = 15; GP2: *n* = 14; GP3: *n* = 10). Beginning from Day 30 after cell injection the signal showed a moderate increasing trend indicative for of tumor growth. Small differences can be seen between the treatment groups but no significant difference in bioluminescence was observed. During the treatment period (Day 16–Day 44) no effect on tumor regression or prevention of metastasis was noted.

After sacrificing the animals, a complete necropsy was performed and documented by photos. Before sacrificing the animals, they were imaged by IVIS Spectrum to identify bioluminescent tissues for explantation. However, comparing the treated groups with the control group, showed no effect of the treatment on tumor regression (Figure 4A). As can be seen in (Figure 4B), the graphs for GP1 (vehicle) and GP2 (LY × OS) and GP3 (LY × IV) show no significant difference in bioluminescence.

**Figure 5:**
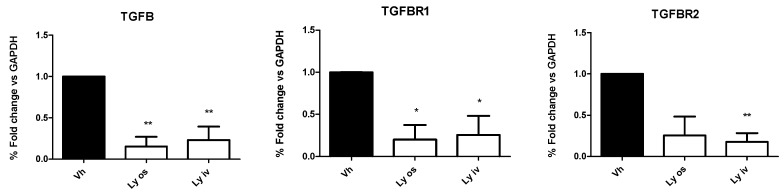
mRNA expression of TGF-β1, TGF-β RI and TGF-β RII investigated by qRT-PCR was significantly down-regulated (* *p* < 0.05; ** *p* < 0.01) in mice treated either orally with nanoparticle encapsulated Galunisertib (Ly os) or intravenously with the Galunisertib solution (Ly iv) as compared to controls (Vh).

In contrast to the in vivo data, in tumor tissues the mRNA expression of TGF-βI, TGF-β RI and TGF-β RII levels, investigated by qRT-PCR, showed a significant (*p* < 0.05) down-regulation in mice treated orally with encapsulated Galunisertib or Galunisertib solution as compared to controls (Figure 5). No difference was found comparing the two Galunisertib formulations.

With this study, we demonstrated that by effectively inhibiting the TGF-β pathway with Galunisertib we observed a statistically significant reduction (*p* = 0.03) of the mRNA level of the drug target compared to the controls treated with vehicle. Nevertheless, this did not affect tumoral growth and progression. A possible explanation of the absence of Galunisertib efficacy can be the cross-talk between tumor and stroma which is hampered by a defective cell immunosurveillance cell and different composition of the microenvironment.

Normally, in the liver there are a multitude of innate and adaptive immune cells, including macrophages, natural killer cells (NK), NK T cells (NKT) and CD8 T cells + CD4 + T cells. Activators of the cellular and humoral immune response are dendritic cells (DC), a specialized family of antigen-presenting cells (APCs) that can also activate NK cells and NKT cells [47] These cells express high levels of cytokines or immunoregulatory factors that induce Treg differentiation, thus helping cancer cells to avoid immune defenses [48,49]. However, several groups found that in patients with cancer including HCC there was a strong reduction in DC levels in peripheral blood allowing cancer cells to escape from the immune system [50,51,52].

More recently, TGF-β involvement in the immune environment has been emphasized, contributing to tumor progression [53]. The Smad2/3 TGF-β pathway critically regulates immune cells in the HCC suppressing CD8+ T cells, natural killer (NK) cells, and dendritic cells (DC), and promotes the development of Treg cells upregulating the transcription factor FoxP3 typically expressed on Treg cells. Patients with HCC show elevated levels of FoxP3 + Treg cells in peripheral blood and a marked increase in tumor-infiltrating Treg cells [54]. Further, Treg CD4 + CD25 + are more present in HCC tissues than CD8 + T cells, most present in peritumoral tissue. Leone et al. in patients with multiple myeloma have found that two distinct, but interdependent populations of CD8 + T cells coexist in the bone marrow. The first population is stimulated by DC, produces IFN-γ and exerts antitumor activity, the second population is stimulated by endothelial cells specifically for the antigen, produces IL-10 and TGF-β and exerts pro-tumor activity, negatively regulating the activity of the first population. Additionally, CD4 + CD25 + Treg cells secrete TGF-β and IL-10 to suppress effector T cells such as CD8 + cytotoxic T lymphocytes infiltrating the tumor into HCC secreting anti-tumor effector molecules such as IFN -γ, IL-2 and TNFα [55,56].

In addition, in the pancreatic ductal adenocarcinoma Argentiero et al. have identified a tumor propensity to educate immune surveillance by reducing the CD8+ and CD4+ effector T cells. Furthermore, they identified a cytokine suppressor profile downregulated in pancreatic ductal adenocarcinoma patients and a relationship between WNT2 overexpression and an immuno-permissive tumor environment [57]. These studies have shown that a low number of intratumoral CD8 + T cells and a large number of regulatory T cells are associated with a poorer prognosis in patients with HCC [58] and this stimulates the use of therapies that could re-educate an immune-reactive tumor environment, within the cancer environment, with a homeostatic immune loss.

TGF-β1 participates also directly in the immune-checkpoint regulation by improving transcriptional expression of the PD-1 highly expressed on exhausted CD8 + T cells through the SMAD3 transcriptional activation. Its interaction with programmed death ligand-1 and 2 (PD-L1/ 2), present on APCs or tumor cells, compromises the cytotoxic function of CD8+ T cells in human HCC and contributes to chemoresistance. These data underline the immunosuppressive role for TGF-β during the HCC progression [59].

However, it was observed in the study by Mariathasan S et al. Tauriello DVF et al., using murine models that exhibited an immune-excluded phenotype, that co-administration of TGF-β and PDL1 blocking antibodies, as compared to monotherapies, reduced TGF-β signaling facilitating T cell penetration in tumors and provoked a robust anti-tumor immunity which led to regression of the tumor. The antitumor benefit observed was verified with greater expression of genes indicative of immune activation [60,61]. These results suggest that clinical co-administration of TGF-β and PDL1 blocking agents may provide more favorable outcomes [62]. In fact, there is an ongoing a clinical study enrolling patients with advanced HCC to study the effect of Galunisertib plus anti-PD-1 drugs (NCT02423343) on improving the antitumor immunity in patients with HCC.

In HCC, the increase in inflammatory cytokines leads to the recruitment of macrophages, through the secretion of VEGF, PlGF, PDGF, TGF-β and glypican-3. These are called tumor-associated macrophages (TAM) and are the main actors of cancer-related inflammation since they can infiltrate the tumor environment (Figure 2). TAMs exert a pro-tumor effect, inhibiting antitumor activities by secreting chemokines such as CCL17, CCL18 and CCL22, which attract Tregs cells to tumor sites, compromising the immune responses of CD8 + cytotoxic T cells [63]. TAMs, are also able to produce pro-angiogenic factors, such as VEGF, PDGF and TGF- β. TAMs also induce angiogenesis expressing matrix metalloproteinases [63,64]. However, regardless of immune surveillance, the increase in VEGF, a key factor in the regulation of angiogenesis, is also secreted by tumor cells and contributes to the formation of new vessels. More precisely, the progenitor cells, attracted by VEGF, migrate through the peripheral blood from the bone marrow to the site of neovascularization of the tumor. This abundant vascularization thus brings more oxygen and nutrients that support tumor growth and metastasis [65].

In addition to VEGF, another crucial growth factor for tumor development is EGF [66]. Kathleen et al. [67] found that primary endothelial cells cultured with conditioned culture of squamous cell carcinoma showed an up-regulation of IL-6, CXCL8 or EGF through STAT3/Akt/ERK signaling. Further studies have also shown that the functional activities of growth factors such as VEGF and EGF are dynamically and mutually controlled by integrins which are involved in signal transduction during angiogenesis by stimulating the assembly of intracellular signaling molecules, such as FAK or integrin bound kinase. These findings reveal a further cross-talk mechanism between the endothelial cells and HCC cells that stimulates the tumor growth [66].

In this context, our study helps explain how the surrounding microenvironment influences the development and progression of fibrosis and cancer and is extremely fundamental for studying the pleiotropic actions of TGF-β. Furthermore, this encourages testing the combination of conventional tumor immunotherapies through vascular targeting treatments.

### 1.5. Exosomes-Moderator of Cross-Talk between Tumor Cells and Microenvironment of HCC

While the role of soluble TGF-β as pleiotropic factor is well defined, exosomes and their influence on the well-orchestrated interplay of TGF-β with the tumor microenvironment, the immune cells, and here in particular with CAFs is still not well understood. However, the role of exosomes in intercellular communication even over long distances is now undoubted. In the following we will show exemplary exosomes with three different cargoes and how diverse the influence of exosomes on the moderation of the TGF-β pathway and its influence on the microenvironment can be.

Exosomes are small membrane vesicles that range in size from 30 to 130 nm in diameter which are produced in the endosomal compartment from multivesicular bodies. They are released in the extracellular milieu under physiological and pathological conditions. Exosomes contain mRNA, long non-coding RNA, protein, DNA and miRNA in a well-defined composition. Experimental proof indicated that the exosomes play a crucial role in development of diseases and in particular in the progress of tumors by influencing the tumor microenvironment as well as preparing the niche for metastasis. In HCC, the tumor is composed of malignant hepatocytes, tumor stromal cells which are activated hepatic stellate cells, cancer-associated fibroblasts, myofibroblasts and immune cells and the ECM and here in particular the development of fibrosis, excessive ECM [68].

**Figure 6:**
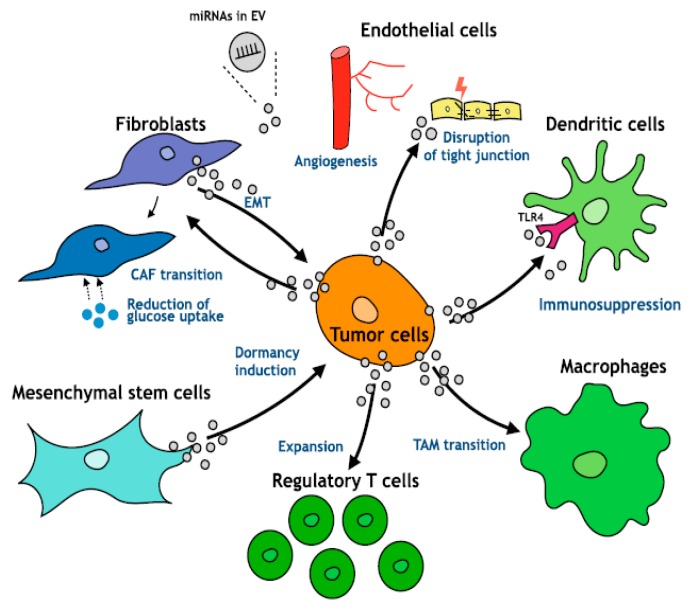
Exosomal miRNAs and the cross-talk in tumor environment. Under Creative Commons Attribution (CC BY) license from: Kogure et al. [72].

Moreover, TAMs—a major component of tumor-infiltrating leukocytes—play an important role in tumor progression (Figure 6) as they acquire an M2-polarized phenotype and promote angiogenesis, metastasis, and suppression of adaptive immunity through the expression of cytokines, chemokines, growth factors, and matrix metalloproteases [69,70]. This transition has an important impact on the TGF-β pathway. Even more importantly, there is a strong difference in TGF-β release from macrophages upon activation by either lipopolysaccharides or INF-y between immune competent “normal” mouse strains such as BALB/C and C57BL/6 mice [71]. M2 macrophages which are more prominent in BALB/C mice than in C57BL/6 mice release a higher amount of TGF-β.

In the following we will focus on the role of exosomal moderated cross-talk between hepatocarcinoma cells and its direct microenvironment and how it is influenced by TGF-β or influences the action of TGF-β. A review by Caja et al. [7] highlights in detail the influence of TGF-β as soluble factor on the different cells of the microenvironment of the HCC and its role in the progression of tumor growth and metastasis and underlines the storage capacity of the extracellular matrix for TGF-β. However, it has to be noted that TGF-β is delivered in a much less controlled manner if it is encapsulated in exosomes and enters directly into target cells. Goulet et al. showed that exosomes derived from bladder cancer cell lines contain a high amount of TGF-β in some cell lines the content in exosomes is significantly higher than the free TGF-β [73]. The exosome-enclosed TGF-β is equally involved in the transition of fibroblast into CAFs as it is the free TGF-β but is not subject to the regulations governed by free, ECM-bound TGF-β.

It has to be noted that exosomes are also a transporter for mRNA or miRNA. Kogure et al. [74] analyzed the miRNA content of HCC cell line derived exosomes and determined that the major target of these exosomes is the activation of the transforming growth factor beta activated kinase-1 (TAK-1) pathway which interacts with the TGF-β receptor-associated factor and contributes to tumor cell invasion. Additionally, the tumor-derived miRNA containing exosomes also play a crucial role in development of lung metastasis. One example is the miR-1247-3p released by highly metastatic HCC which induces the fibroblast-CAF transition even in distant sites such as the lung and hence participates in preparing the metastatic niche. Similar effects of exosomal miR-1247-3p occur in mice and in patient’s tissue [75].

Another role of exosomes in the TGF-β pathway was observed by Fricke et al. [73] who investigated colorectal cancers lacking DNA mismatch repair function and therefore prone to accumulate frameshift mutations at short repetitive DNA sequences (microsatellites). It turns out that the DNA mutations especially involving the TGF-β RII cMNR frameshift mutations are transferred by DNA in exosomes. The uptake of these DNA containing exosomes by recipient cells in consequence modifies both their corresponding exosome proteome and cytokine release profile. This mechanism needs further investigation for a potential role in HCC.

These three examples for different exosomal cargoes underline how exosomal delivered content such as proteins, miRNA and even DNA fragments can contribute to the dysregulation of the well-orchestrated TGF-β pathway and hence the progression of cancer or development of metastasis. The different targets in the tumor environment such as fibroblast-CAF transition and influence of the EMT of cancer cells or modification of cells by DNA frameshift fragments support the importance of proper animal models for potential drug testing as well as to understand the mechanisms involved in development and progression of cancer.

As we have seen different animals are used to study HCC. One can mainly distinguish two models, immune competent and immune deficient mice. Immune competent mouse models such as Alb-Cre Tg or ELF knockout which are genetically modified mice as well asB6C3F1 mice injected with a single dose of DEN or C57BL/6 mice receiving hydrodynamic tail-vein injections of HRASG12V and a short hairpin RNA that downregulates p53, HRASG12V and MYC, and HRASG12V and TAZS89A develop spontaneous orthotopic HCC that mimic very closely the conditions found in human tumors in terms of the tumor specific microenvironment and TGF-β moderated cross-talk with the cells of the immune system and in the microenvironment such as CAF.

In contrast, immune deficient mice (Female BALB/c-nu/nu or BALB/C nude mice, see Table 1) are often used to transplant either cells or microtumors grown from cell culture of cell lines or even patient-derived tumors. Here different factors have to be taken into consideration which can influence either the microenvironment of the tumor or the exosomal exchange of material. In nude mice macrophages and dendritic cells are still present while mature T cells are absent as can be seen in (Table 5) while in other mouse models even those are defective. These limitations are especially important for models based on injected tumor cells from cell lines in which other cells than the human tumor cells are absent and the developing tumor in the injection site has to accumulate and modify the immune cells and fibroblasts from the host organism (mouse).

Transplanted tumor tissue from the patient on the other hand comes with a fully developed microenvironment which then needs to cross-talk with the mouse microenvironment. As can be seen in (Table 2), at least for NOD scid gamma mouse models it is known that they are maintaining the human microenvironment after transplantation of the xenograft. Some few studies already documented that inter-species miRNA exchange is possible such as epithelial cell-gut microbiota cross-talk or plant-bacteria, -nematode, or -fungus information exchange [52] and also some exosomal miRNA such as miR-214 works in humans and in mice the same way [53] Moreover, miR-214 is also known to play an pivotal role in tumor suppression and is considered to be a potential therapeutic exosomes for treatment of liver cancer [54]. Interestingly, these exosomes are released from human liver stem cells.

In summary it can be concluded that the role of exosomes in the cross-talk between tumor cells, their microenvironment and distant metastatic site needs to be considered especially if therapeutic failure for the targeted treatment of the TGF-β pathway is observed. For example, the protected status of the TGF-β enclosed in exosomes may allow the tumor to bypass the control of inhibitors. The exosomes contribute a complex toolbox both for tumor-suppressive and tumor-progressive activities and should be studied in detail along with the proteomic and genomic changes induced by the tumor cells to the microenvironmental cells and vice versa. These aspects need also proper consideration when choosing the right animal model.

## 2. Conclusions

In order to translate drug development from bench-to-bedside fast and reliably, animal models need to be predictive for the drug efficacy in humans. Therefore, it is important that the animal model mimics the complexity of a spontaneous tumor, the immune system and the microenvironment. Recently the influence of the microenvironment on tumor growth, progression and metastasis was highlighted. In this context, especially the role of TGF-β which can act both as a tumor-suppressor or tumor-promoter and is released by tumor cells as well as by myofibroblasts or CAFs is elevated importance as it moderates between the malignant cells, the cells of tumor environment and immune system. In particular, in HCC, a tumor which is highly depended on microenvironmental conditions such as fibrosis and invading macrophages and fibroblasts this cross-talk between tumor and stromal cells needs to be taken into consideration by the chosen animal model. While simplified systems such as injection of tumor cell lines under the skin of the flank of a mouse allow an easy access to the tumor site, small changes in the protocol such as moving the implantation site from flank to paw can lead to a more realistic model and therefore mimics better the conditions in human patients and spontaneous tumors. Future genetically modified mouse models will be even more complex as they need not only take into consideration the interaction of soluble factors with tumor and stromal cells but also the more complex communication via extracellular vesicular payload.

## Figures and Tables

**Table 1:** Animal models for the role of transforming growth factor beta in hepatocellular carcinoma.

Mouse Strain	Immune Competence	Tumor Formation	Model	Aim of the Study	Results	Reference
C57BL/6	Competent	Induced	Hydrodynamic tail-vein injection with HRASG12V	Overexpression of SMAD7 or knockdown of SMAD2,3,4 and its influence on TGF-β pathways	TGF-β inhibition reduced formation and growth of liver tumors when RAS, TAZ proteins and short hairpin RNA are expressed	[40]
B6C3F1	Competent	Induced	Injection of DEN. Single injection of TGF-β before sacrifice	Evaluation of liver apoptosis extent by exogenous TGF-β	Apoptosis is high in HCC and increases even more by administration of pro-apoptotic cytokine	[41]
ELF (embryonic liver fodrin) knockout	Competent	Spontaneous	ELF knockouts develop HCC in 15 months	ELF as a target for enhancing TGF-β pathway to suppress tumor formation	Loss of ELF causes disruption of TGF-β pathways and HCC development	[42]
Tak1ΔHep	Competent	Spontaneous	Tak1ΔHep mice develop HCC in 9 months	TGF-β signaling in TAK1 deleted hepatocytes	TGF-β promotes HCC and expression of anti-apoptotic, pro-oncogenic, and angiogenic factors	[43]
Female BALB/C nude	Deficient	Induced	Subcutaneous injection with Hg2 cells	Effects of sulforaphane on TGF-β pathways	Sulforaphane inhibits TGF-β linked EMT transition	[44]
Female BALB/C nu/nu	Deficient	Induced	Oral administration of EW-7197 (ALK 5 inhibitor) in orthotopic model/implanted SK-HEP1-Luc cells	ALK 5 inhibition effects TGF-β signaling between Stellate cells and HCC cells	ALK 5 inhibitor interferes with tumor growth	[45]

DEN: N-nitrosodiethylamine.

**Table 2:** Recovery of Galunisertib following the method of ratio of slopes between Standards (STDs) in solution and plasma.

Samples	Intercept	Slope	R%
STDs Solution	8.65	224.73	99.94
STDs Plasma	6.60	201.91	99.95
	Recovery%	89.80	

**Table 3:** Linearity, precision and accuracy of the method for analysis of Galunisertib in mouse plasma samples.

Concentration	0.05	0.1	0.25	0.5	1	2.00	Intercept	Slope	R%
DAY1	0.055	0.085	0.294	0.505	0.998	1.957	2.54	207.38	98.31
DAY2	0.044	0.076	0.269	0.509	1.011	1.991	6.60	201.91	99.95
DAY3	0.053	0.090	0.286	0.495	0.992	1.962	3.19	205.36	99.16
Mean	0.05	0.08	0.28	0.50	1.00	1.97	4.11	204.88	99.14
SD	0.006	0.007	0.013	0.007	0.010	0.018		2.77	0.82
Precision	11.565	8.480	4.511	1.434	0.971	0.932			0.83
Accuracy	1.333	−16.333	13.200	0.600	0.033	−1.500			

SD: standard deviation.

**Table 4:** Pharmacokinetic parameters of Galunisertib in mice following a single oral dose of two different formulations.

Parameters	Suspension (75mg/kg)	Solution (150 mg/kg)
K_elim_ (h^−1^)	0.49	0.59
T_1/2_ (h)	1.40	1.71
C_max_ (µg/mL)	3.33	19.01
T_max_ (h)	0.5	0.5
AUC_last_ (µg/h/mL)	3.11	15.48
Relative bioavailability (F)		2.49

K_elim_: elimination rate constant; T1/2: half-life_;_ C_max:_ maximal concentration; AUC_last:_ Area under the curve.

**Table 5:** Tumor- and chronic inflammation-relevant immune cells in different immunodeficient mouse models [51].

	Mouse Model	NSG™	NRG	NSGS	NOD *scid*	BALB *scid*	B6 *Rag1*	Nude
Immune Cells								
Macrophages		defective	defective	defective	defective	present	present	present
Dendritic cells		defective	defective	defective	defective	present	present	present
Mature T-cells		absent	absent	absent	absent	absent	absent	absent
note		Capable of maintaining a human tumor microenvironment after engraftment						

NOD: Non-obese diabetic; scid: Severe Combined Immunodeficiency; NSG: NOD scid gamma; NRG: NOD *Rag* gamma; NSGS: NOD *scid* Gamma Il3- GM-SF (NSG-SGM3).

## Abbreviations

CAFs	cancer-associated fibroblasts
DCs	dendritic cells
DEN	N-nitrosodiethylamine
ECM	extracellular matrix
EGF	epidermal growth factor
ELF	embryonic liver fodrin
EMT	epithelial-mesenchymal transition
FAK	focal adhesion *kinase*
FGF	fibroblast growth factor
HCC	hepatocellular carcinoma
HGF	hepatocyte growth factor
HSC	hepatic stellate cells
IFN-γ	interferon-gamma
IFN	interferons
IL	interleukins
miRNAs	microRNAs
NK	natural killer cells
NKT	natural killer T cells
NOG mice	NOD/Shi-Scid/IL-2Rγ ^null^
PDGF	platelet-derived growth factor
PD-L1/ 2	programmed death ligand-1 and 2
SDF	stromal-derived factor
TAM	tumor-associated macrophages
TGF	transforming growth factor
TGFβR1	Type I dimeric receptor
TGFβR2	Type II serine/threonine kinase dimeric receptor
TNF-α	tumor necrosis factor-α
Treg	regulatory T
VEGF	vascular endothelial growth factor
α-SMA	α smooth muscle actin
DMSO	dimethylsulfoxide
PEG	polyethylene glycol
EtOH	ethanol
